# Classification and characterisation of brain network changes in chronic back pain: A multicenter study

**DOI:** 10.12688/wellcomeopenres.14069.2

**Published:** 2018-10-10

**Authors:** Hiroaki Mano, Gopal Kotecha, Kenji Leibnitz, Takashi Matsubara, Christian Sprenger, Aya Nakae, Nicholas Shenker, Masahiko Shibata, Valerie Voon, Wako Yoshida, Michael Lee, Toshio Yanagida, Mitsuo Kawato, Maria Joao Rosa, Ben Seymour

**Affiliations:** 1 Center for Information and Neural Networks, National Institute of Information and Communications Technology, Osaka, Japan; 2 Cambridge University Hospitals NHS Foundation Trust, Cambridge, UK; 3 Graduate School of System Informatics, Kobe University, Kobe, Japan; 4 Computational and Biological Learning Laboratory, Department of Engineering, University of Cambridge, Cambridge, UK; 5 Osaka University School of Medicine, Osaka, Japan; 6 Immunology Frontiers Research Center, Osaka University, Osaka, Japan; 7 School of Clinical Medicine, University of Cambridge, Cambridge, UK; 8 Advanced Telecommunications Research Center International, Kyoto, Japan; 9 Max-Planck UCL Centre for Computational Psychiatry and Ageing Research, University College London, London, UK; 10 Department of Computer Science, University College London, London, UK

**Keywords:** Chronic pain, Nociception, Connectomics, graph theory, deep learning, sensorimotor, multislice modularity, hub disruption, osteoarthritis, arthritis, rostral ACC, endogenous modulation

## Abstract

**Background.** Chronic pain is a common, often disabling condition thought to involve a combination of peripheral and central neurobiological factors. However, the extent and nature of changes in the brain is poorly understood.

**Methods.** We investigated brain network architecture using resting-state fMRI data in chronic back pain patients in the UK and Japan (41 patients, 56 controls), as well as open data from USA. We applied machine learning and deep learning (conditional variational autoencoder architecture) methods to explore classification of patients/controls based on network connectivity. We then studied the network topology of the data, and developed a multislice modularity method to look for consensus evidence of modular reorganisation in chronic back pain.

**Results.** Machine learning and deep learning allowed reliable classification of patients in a third, independent open data set with an accuracy of 63%, with 68% in cross validation of all data. We identified robust evidence of network hub disruption in chronic pain, most consistently with respect to clustering coefficient and betweenness centrality. We found a consensus pattern of modular reorganisation involving extensive, bilateral regions of sensorimotor cortex, and characterised primarily by negative reorganisation - a tendency for sensorimotor cortex nodes to be less inclined to form pairwise modular links with other brain nodes. Furthermore, these regions were found to display increased connectivity with the pregenual anterior cingulate cortex, a region known to be involved in endogenous pain control. In contrast, intraparietal sulcus displayed a propensity towards positive modular reorganisation, suggesting that it might have a role in forming modules associated with the chronic pain state.

**Conclusion.** The results provide evidence of consistent and characteristic brain network changes in chronic pain, characterised primarily by extensive reorganisation of the network architecture of the sensorimotor cortex.

## Introduction

Maladaptive brain processing of pain is thought to have a primary or facilitative role in many types of chronic pain. In chronic back pain, for example, degenerative musculoskeletal change is considered unlikely in itself to fully explain persistent pain in most patients, and central processes are thought to be critical for the chronification and maintenance of pain. Existing data have identified a broad array of structural and functional brain differences in patients ( Baliki *et al.*, 2008; Baliki *et al.*, 2011; Hashmi *et al.*, 2013; Hemington *et al.*, 2016; Kutch *et al.*, 2017; Napadow *et al.*, 2010; Tagliazucchi *et al.*, 2010), and this has led to the concept of chronic pain as a brain network disorder ( Apkarian *et al.*, 2009; Kuner & Flor, 2017; Mano & Seymour, 2015). However, given the complexity of brain networks, we still do not have a reliable and consistent characterisation of these changes.

One of the difficulties in identifying robust changes in brain networks underlying chronic pain is that networks are inherently data-rich, and the patterns of disruption may be complex. One way to tackle this is to use machine learning and deep learning methods, and a number of studies have shown how this can be used to successfully build biomarkers (i.e. classifiers) in a range of psychiatric disease ( Takagi *et al.*, 2017; Watanabe *et al.*, 2017; Yahata *et al.*, 2016; Yamada *et al.*, 2017). However, these methods need to be validated on genuinely independent data sets to be convincing, and current evidence of generalisable classifiers for chronic pain is lacking.

Even so, interpreting brain network changes based purely on classifiers alone can be difficult. This is because the classifier pattern itself is often comprised of a large matrix of individual functional connections, and strongly predictive (i.e. information-rich) functional connections do not necessarily imply an active role in a disease. A better way of describing and understanding networks is to instead evaluate the underlying topology ( Bressler & Menon, 2010; Bullmore & Sporns, 2009). Since the brain is inherently modular, individual differences in function can be reflected by differences in a number of network characteristics ( Meunier *et al.*, 2010). This approach offers a way to define specific aspects of network architecture that change in a disease.

With these issues in mind the aim of the current study was to i) classify, and ii) characterise brain networks in chronic back pain in a multi-site study using resting state fMRI. For classification, we applied machine learning and deep learning classifiers based on data from two sites (Cambridge, UK and Osaka, Japan) as a discovery cohort, and used an open data set (Chicago, USA) as a validation cohort. For characterisation, we investigated hub disruption across all datasets, and developed a method to identify brain regions that undergo modular reorganisation in the chronic pain state.

## Methods

### Participants

We recruited adults with chronic musculoskeletal low back pain (CLBP) and approximately age, sex, and IQ-matched adults without CLBP at two sites: Cambridge, UK (June 2013 – April 2014, 17 patients, 17 controls) and Osaka, Japan (April 2014 – March 2015, 24 patients, 39 controls). Patients were recruited under the following inclusion criteria: chronic back pain for over 6 months, no other chronic pain condition, no other major neurological or psychiatric disease, and no contraindications to MRI scanning. The study was approved by the East of England NRES Committee, Norfolk, UK (reference 13/EE/0098); and the Ethics Committee for Human and Animal Research of the National Institute of Information and Communications Technology, Japan (reference 20140611). Prior to the participation, all participants gave written informed consent.

For all participants, the pain scores were taken in the form of visual analog scale (VAS) and Short-Form McGill Pain Questionnaire. Mood information was collected with Beck Depression Inventory (BDI) and Hamilton Depression Rating Scores. IQ information was collected using the National Adult Reading Test (NART) for the participants in the UK, and the Japanese Adult Reading Test (JART) for the participants in Japan. Demographic information is summarised in Table 1.

**Table 1.** Demographic details of participants.

		*N*	Age	BDI	Duration	VAS	JART/NART
CLBP	JP	24	21–66	15.2 ± 10.5	11.6 ± 9.2	2.6 ± 2.4	31.23 ± 9.25
	UK	17	20–61	15.9 ± 11.5	10.4 ± 7.5	4.8 ± 2.8	29.31 ± 6.76
	US	34	21–62	6.3 ± 5.8	15.7 ± 11.3	6.7 ± 1.7	— —
TD	JP	39	21–68	4.7 ± 3.4	0	0.3 ± 1.1	34.66 ± 7.38
	UK	17	20–62	3.7 ± 5.3	2.4 ± 7.5	0.3 ± 0.7	37.29 ± 6.84
	US	34	21–64	1.5 ± 2.6	0	0	— —

We used an openly available US data set ( OpenPain Project, Department of Physiology, Northwestern University) to provide an additional validation sample for classification, and to add to the data used for network characterisation. For details, see the *Data availability* section below.

### MRI data acquisition

All the scans were performed on a 3.0-T MRI Scanner (3T Magnetom Trio with TIM system; Siemens, Erlangen, Germany) equipped with echo planar imaging (EPI) capability and a standard 12-channel phased array head coil either at Addenbrooke’s hospital (Cambridge, UK) or CiNet (Osaka, Japan). Participants remained supine and wore MR-compatible headphones with their heads immobililised with cushioned supports during scanning. Resting-state functional MRI (rsfMRI) was acquired using a single-shot EPI gradient echo T2*-weighted pulse sequence with the following parameters: for the participants in the UK - TR 2000 ms, TE = 30 ms, FA = 78 degrees, BW = 2442 Hz, FOV = 192 × 192 mm (covering the whole brain), acquisition matrix = 64 × 64, 32 axial slices with a interleaved slice order of 3.0mm slice thickness with 0.75mm inter-slice gap, 300 volumes; for the participants in Japan - TR 2500 ms, TE = 30 ms, FA = 80 degrees, BW = 2367 Hz, FOV = 212 × 212 mm (covering the whole brain), acquisition matrix = 64 × 64, 41 axial slices with an ascending slice order of 3.2mm slice thickness with 0.8mm inter-slice gap, 234 volumes. A high-resolution three-dimensional volumetric acquisition of a T1-weighted structural MRI scan was collected using a MPRAGE pulse sequence: for the participants in the UK - TR = 2300 ms, TE = 2.98 ms, time of inversion = 900 ms, FA = 9 degrees, BW = 240 Hz, FOV = 256 × 256 mm, 176 sagittal slices of 1mm slice thickness with no inter-slice gap, acquisition matrix = 256 × 256; for the participants in Japan - TR = 2250 ms, TE = 3.06 ms, time of inversion = 900 ms, FA = 9 degrees, BW = 230 Hz, FOV = 256 × 256 mm, 208 sagittal slices of 1mm slice thickness with no inter-slice gap, acquisition matrix = 256 × 256.

### Resting-state fMRI data preprocessing

High-resolution T1-weighted anatomical imaging and a resting-state functional imaging were performed for each participant, and all those images were preprocessed with SPM8 (Wellcome Trust Centre for Neuroimaging, University College London, UK) on Matlab (R2014a, Mathworks, USA). The first five volumes were discarded to allow for T1 equilibration. Slice timing was adjusted to the intermediate slice and all images were realigned to the first volume of each scan with an estimation based on all the voxels, a fine sampling distance of 2 mm, and a 7th order B-spline interpolation to ensure an accurate head motion correction with the estimated 6 rigid-body head motion parameters. After T1 weighted structural image was co-registered to the mean EPI volume, tissue segmentation of the structural image into three tissue classes; gray matter (GM), white matter (WM), and cerebrospinal fluid (CSF), based on the T1-weighted image contrast was performed in the common Montreal Neurological Institute (MNI) space. The relevant parameters estimated in the tissue segmentation were applied to warp functional images into MNI152 template space with a 2 × 2 × 2 mm spatial resolution. Subsequently, smoothing was applied with a 6 × 6 × 6 mm FWHM Gaussian kernel.

### Inter-regional correlation analysis

To investigate the inter-regional functional relationship among regions over the whole brain, we used the digital BSA-AAL composite atlas composed of 140 ROIs consisting of the BrainVISA Sulci Atlas (BSA) and the Anatomical Automatic Labeling (AAL) package (with a spatial resampling of 2 × 2 × 2 mm ^3^ grid in MNI space); a band-pass filter with a transmission range from 0.008 to 0.1 Hz; regression out of the nuisance regressors from a mask of white matter, cerebrospinal fluid, and the whole brain based on the segmentation of individual T1 weighted image, and three translational and three rotational head motion parameters. To protect against motion artifact in inter-regional correlation, we performed scrubbing ( Power *et al.*, 2012) in which any frames exhibiting abrupt and excessive head motions were identified with a threshold of frame displacement (FD) of 0.5 mm, and all the frames identified were removed from individual time-series, along with the previous one and the two successive frames. This preserved mean proportion of 91.7% and 85.0% of slices frames in controls and CLBP patients in the Japan data respectively, 81.3% and 75.4% in the UK data, and 93.5% and 88.8% in the US data, with no significant difference (difference in mean proportion of the remained frames; CI; p-value is as follows: 6.70, 95%; -1.8218 to 15.1891; P = 0.1213 in the Japan data, 5.90, 95%; -10.4544 to 22.1636; P = 0.4696 in the UK data, 4.70, 95%; -1.4705 to 10.8421; P = 0.1331 in the US data), and no difference between controls and CLBP patients was observed in mean FD in the remained frames at any site with a bootstrap estimation of 95% confidence interval (mean FD difference: -0.016; Bootstrap 95% CI: -0.054 to 0.023 in the Japan data, Mean FD difference: -0.029; Bootstrap 95% CI: -0.061 to 0.003 in the UK data, Mean FD difference: -0.022; Bootstrap 95% CI: -0.059 to 0.015 in the US data; if a confidence interval that does not span zero can be taken to imply a significant difference (p<0.05).

A patient in the UK data was excluded from further analysis by an exclusion criteria that the root mean squared change in BOLD signal from volume to volume (DVARS) after the scrubbing showed more than 3 interquartile ranges above the upper quartile or below the lower quartile. Subsequently a 140 × 140 Pearson’s full correlation matrix was computed on all pairs of each of intra-regional average time-series of the ROIs.

### Classification

A classification model built from UK and Japan data sets was tested on an open data set available from the “ OpenPain Project” (Department of Physiology, Northwestern University). Anatomical MRI data in the test data set were provided with skull stripping during preprocessing. We chose to exclude six participants (three CLBP patients and three controls) from the US validation test set that had lost a small part of brain coverage in their anatomical image during the skull stripping procedure. Note that the US dataset differed in the exclusion of patients with a BDI score of over 19.

### Classification using Support Vector Machines

We used a Support Vector Machine (SVM) classifier ( Cortes & Vapnik, 1995) based on the connectivity (correlation) matrices to classify subjects as patient or control. SVMs learn a hyperplane, or decision boundary, that separates the two classes as well as possible (i.e. maximises the margin between the samples in the two classes). Once this boundary is learnt new samples are classified according to the side of the hyperplane they fall. The optimal margin is parameterised by a weight vector, *W*. Each entry of *W* corresponds to a particular feature, in this case a connectivity measure between two brain regions, and is interpreted as the contribution of the feature to separating the classes. However, it is important to note that the predictions are based on all features. Linear kernel SVMs have only one hyperparameter, *C*, controlling the trade-off between the width of the margin separating the two classes and the number of misclassified samples. To assess the predictive performance of the SVM classifier we ran two validation models: i) pooling together the UK and Japan data as the training dataset, and using the US data as an independent validation dataset ( *validation model 1*); ii) pooling the three datasets together (UK, Japan and US) to increase power and testing the performance of the classifier using a stratified Leave-Two-Subjects-Out (LTSO) cross-validation (CV) ( *validation model 2*). LTSO CV allowed for one subject of each class to be left out for testing, and the remaining subjects from both classes to be used for training in each CV fold. To account for multi-site effects, the pairs that were left out were always from the same acquisition site (stratification).

Due to the slightly higher number of controls compared to patients, we also bootstrapped 100 models in both validation approaches. In other words, each time we ran the whole validation model we randomly selected a balanced sample (as large as possible) with an equal number of patients and controls. The predictive accuracies were averaged across bootstraps.

Feature selection was carried out on the training data using a univariate two-sample t-test ( Guyon & Elisseeff, 2003): we Fisher-transformed the correlation data and kept only the features (connections) statistically significant between patients and controls ( *p* < 0.05, uncorrected).

For both validation approaches the SVM *C* parameter was optimised using grid search (between 10 ^–3^ to 10 ^3^) and a Leave-3-Out CV on the training data. This CV was nested within the LTSO CV in the second validation model.

We used as performance measures the accuracy (percentage of correctly classified samples) and both sensitivity and specificity (percentage of correctly classified patients and controls, respectively). The obtained results were tested for statistical significance (i.e. how unlikely the results would be if the classifier was randomly attributing the class labels) using a permutation approach, where we repeat the entire classification procedure (including the two validation models, parameter optimisation, bootstrapping and feature selection) 1, 000 times, each time permuting the labels (patient or control) ( Nichols & Holmes, 2002).

We used *python* 2.7.12 and the *scikit-learn* 0.17.1 machine learning library ( Pedregosa *et al.*, 2011) for this analysis.

### Classification using Deep Learning

We used a conditional variational autoencoder (CVAE) based on the 140 ROIs to classify subjects ( Kingma *et al.*, 2014; Tashiro *et al.*, 2017). A CVAEs is a generative probabilistic model based on multilayer neural networks. Given an input data *x* and a condition *y*, a CVAE builds a model of the conditional probability log *p*( *x*| *y*). We used a CVAE as a classifier based on log-likelihood. We emphasize that the CVAE is not based on the 140 × 140 Pearson’s full correlation matrices but is based on 140-dimensional vectors, each corresponding to the intra-regional average signal intensities of the 140 ROIs at one time point. Let $x_{j}^{\left(i\right)}$ denote the signal intensity of the *i*-th ROI obtained from a subject at time point *j*. Each sample is a 140-dimensional vector: *x _j_* = $x_{j}^{\left(1\right)}$,… $x_{j}^{\left(140\right)}$) ^*T*^ and all the samples obtained from a subject is represented by the set *X* = { *x _j_*}. The CVAE consists of two neural networks called encoder and decoder. The encoder accepts a sample *x _j_* and the condition *y* of a subject, and infers a posterior probability of the latent variable *z _j_*, which is considered to correspond to a nuisance component unrelated to the disease such as something comes into the subject’s mind at the time point *j*. The decoder accepts the latent variable *z _j_* and the condition *y*, and generates an artificial sample *x̃ _j_*. After training the encoder and decoder jointly, the CVAE reconstructs given signal *x _j_* under the condition *y* accurately, and the reconstruction error indicates (an upper bound of) the negative log-likelihood – log *p*( *x _j_*| *y*) of the given signal *x _j_* (see the original study ( Kingma *et al.*, 2014) for details). Since we considered that each sample *x _j_* was sampled independently from each other, the log-likelihood log *p*( *X*| *y*) of all the samples *X* of a subject was equal to the summation of the log-likelihood of each sample, i.e., log *p*( *X*| *y*) = ∑ _*j*_ log *p*( *x _j_*| *y*).

Given the samples *X*, the posterior probability *p*( *y*| *X*) that the subject belongs to the class *y* is assumed as *p*( *y*| *X*) = *p*( *X*| *y*) *p*( *y*)/ *p*( *X*) ∝ *p*( *X*| *y*) *p*( *y*) according to Bayes’ theorem. Therefore, *p*( *X*| *y* = 1) *p*( *y* = 1) > *p*( *X*| *y* = 0) *p*( *y* = 0) indicates that the subject is classified into the class *y* = 1. Finally, we assumed that *p*( *y* = 1) = *p*( *y* = 0) = 0.5 for adjusting the imbalance.

We used the encoder and the decoder consisting of 4 layers: The number of units were denoted by *n*_0_, *n*_1_, *n*_2_, and *n*_3_. The hidden layers employed ReLU and layer normalization as their activation functions, and the output layers employed identity function. The condition *y* was represented by the bias terms of the second hidden layer of the encoder and the first hidden layer of the decoder. The CVAE was trained by Adam optimization algorithm with the parameter α = 10 ^–3^, β _1_ = 0.9, and β _2_ = 0.999. For each learning iteration, 10 patients and 10 controls were randomly chosen from each site, and 50 samples were randomly chosen from each of the chosen subjects, indicating that a mini-batch comprised 2000 samples. The number of units were searched for within the ranges of *n*_0_ = 140, *n*_1_ ∈ {50, 100, 200}, *n*_2_ ∈ {50, 100, 200}, and *n*_3_ ∈ {5, 10} using a Leave-Four-Subjects-Per-Group-Out CV for *validation model 1* and a 10-fold CV for *validation model 2*, where the 10-fold CV was nested within the LTSO CV. We also used *python* 2.7.12 for this analysis.

We can obtain a marginal log-likelihood log *p*( $x_{j}^{\left(i\right)}$| *y*) of the *i*-th ROI at the time point *j* using the trained CVAE. A large difference between the marginal log-likelihood log *p*( $x_{j}^{\left(i\right)}$| *y*) given the different class labels *y* = 0 and *y* = 1 indicates that the *i*-th ROI largely contributes to the classification. Hence, we defined ℒ *_i_* =∑ _*j*_ log *p*( $x_{j}^{\left(i\right)}$}| *y* = *c*) – log *p*( $x_{j}^{\left(i\right)}$}| *y* = 1 – *c*)) given the correct label *c* as the contribution weight of the *i*-th ROI.

### Characterisation of network changes: Hub Disruption

To study the topology of brain networks, we thresholded the Pearson’s full correlation matrices to a produce binary adjacency matrix (consisting of 1’s and 0’s) for each subject. Each of the correlation matrices was thresholded in an adaptive manner to produce an adjacency matrix with a 10 % link density. This value was chosen based on previous studies that have found such a link density to provide optimal discriminative ability ( Achard *et al.*, 2012; Itahashi *et al.*, 2014; Mansour *et al.*, 2016; Termenon *et al.*, 2016). Using the adjacency matrices, we calculated the Hub Disruption Index (HDI) - a well-recognized method that characterises functional reorganisation in resting-state brain networks in disease ( Achard *et al.*, 2012; Itahashi *et al.*, 2014; Mansour *et al.*, 2016; Termenon *et al.*, 2016). HDI is calculated based on the difference in a nodal graph-theoretic property of the network, and references the distribution of this metric across all nodes in a single subject, in comparison to the equivalent referential distribution in a cohort of healthy controls. Nodal degree is the most-used index, but any nodal graph measure can be used. Using the Brain Connectivity Toolbox ( Rubinov & Sporns, 2010), we examined HDIs for degree, clustering coefficient, betweenness centrality, eigenvector centrality, K-coreness, flow Coefficient, local efficiency, and participation coefficient, and present results for those measures that were consistently significant across all data-sets. This choice is based on the measures that have been applied in previous studies ( Achard *et al.*, 2012; Hashmi *et al.*, 2014; Mansour *et al.*, 2016).

Each of the HDIs, defined as a summary of profile of nodal topological metrics in either a patient compared with the cohort of healthy controls or a control out of the cohort of healthy controls compared with the rest of the cohort of healthy controls (in the same manner as leave-one-out cross-validation), were compared between groups by two-sample two-tailed t-tests and the differences were assigned statistical significance at p values less than 0.05. It should be noted that the way HDI is calculated makes it intrinsically susceptible to data noise, which will tend to produce significant values. In addition, because the control group are used to define the normal values against which the patients are compared, we cannot use the individual values for classification, since the values are not indendent (i.e. there is ‘information leak’ between the classification sets).

In response to reviewers’ comments, we also considered if the HDI was robust to either removal of the top 5% of nodes, or random removal of 90% of nodes, which it was (see Supplementary File 1 for further details).

### Characterisation of network changes: Modular Reorganisation

**Figure 1.**
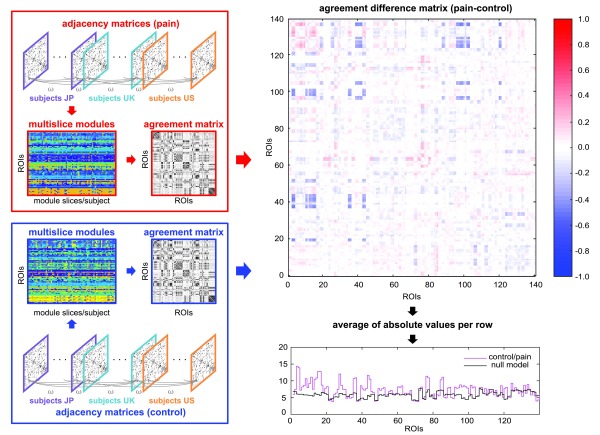
Overview of the computation pipeline for multislice modularity and agreement matrices. First, we calculated the multislice modularity and agreement matrices separately for the pain and control groups, and then calculated their difference. This difference matrix consists of positive values (red) which reflect the likelihood of the two corresponding ROIs (defined by the row and column index) appearing in the same module in the pain group, but not in the control group. Negative values (blue) reflect the opposite - that the two ROIs are less likely to be in the same module in the pain group. Furthermore, values that are near zero (white) reflect pairs of ROIs the do not significantly change or had near-zero agreement in pain and control. The absolute sum of positive and negative values yields an overall metric of modular reorganisation for each ROI (purple plot in lower panel), which can be compared to a chance level calculated from random permutations of the pain and control groups.

In order to study the architecture of brain networks in more depth, to identify (and localise, where possible) key differences between patients and control groups, we next probed the network’s modular structure. Our approach was designed to focus on the differences in brain network modularity between patients and control groups by identifying a measure of the consensus modularity pattern across all of the data. We did this by using a new method based on calculation of the multislice modularity ( Mucha *et al.*, 2010) - which allows estimation of the basic community or modular architecture across large and complex network data sets. In the *categorical* multislice modularity algorithm ( Jeub *et al.*, 2011), the same node is coupled among all subjects of the same group using a coupling parameter ω to create a single symmetric agreement matrix representing each group, see Figure 1.

This agreement matrix is generated with two free parameters, which we defined *a priori*. First, we chose a modularity resolution of γ* = 1.5, given that this leads to roughly 10–20 modules overall, which is consistent with known architecture of brain networks. Second, we chose a ‘moderate’ coupling strength of ω* = 0.1 based on ( Mucha *et al.*, 2010).

The agreement matrix was estimated across all three data sets, separately for the pain patients and the control groups. Since there are slightly more control ( *n*=87) than pain patients ( *n* = 71), we selected a subset of 70 subjects randomly from each group to match the estimation between each group. Since the modularity estimation is a probabilistic procedure, we repeated this 1000 times, selecting the 70 subjects randomly each time, and computed the average agreement matrix across all repetitions.

We next defined an agreement difference matrix *AD* as the difference of agreement matrices of pain *minus* that of the control group. Since each agreement matrix has entries within [0, 1], large positive entries in *AD* represent those node pairs that have high agreement in pain, i.e., nodes that are frequently in the same modules for the pain group, but not in the same modules in the control group. Similarly, large negative entries indicate the opposite case, i.e., nodes that frequently join the same modules in the control group, but are not in the same module for the pain group. Nodes with agreement differences near 0 indicate that two nodes are either in the same module for both groups or they are in different modules.

Since the agreement difference matrix has both positive and negative entries in each column, we independently summed the positive-valued and negative-valued elements. This permitted computing a profile of the strongest contributing ROIs in both cases. The sum of the positive and negative contributions provides an overall metric of modular reorganisation for each ROI.

To statistically evaluate the modularity of each ROI, we performed an approximate permutation test, in which we mixed and randomly resampled the pain and control subjects into two groups, and repeated the full analysis. We did this also 1000 times, and calculated the one-sided *p*-value based on the proportion of times the resampled modularity reorganisation metric exceeded the value based on the correctly specified groups. These results are presented uncorrected for multiple comparisons (across ROIs) below an arbitrary threshold of *p* < 0.01. However we had prior hypotheses related to the 3 sets of regions commonly implicated in chronic pain: sensorimotor cortices, insular-cingulate cortices, and striatal-medial prefrontal cortex.

### Pregenual ACC connectivity analysis

This was based on the same preprocessing pipeline as above. Voxel-wise maps of connectivity, based on Fisher-transformed correlations of voxel-based BOLD time series, were computed to evaluate regions that were more or less correlated in patients than controls. This was based on a bilateral pgACC seed was a 6mm diameter sphere centered on [+/-3,40,5], based on our recent study identifying this region in endogenous control of persistent pain in healthy subjects ( Zhang *et al*., 2018). Statistical analysis was based on simple t-contrasts.

### VBM analysis

We also considered whether there were grey matter changes between groups. Data were analyzed with SPM12 and Matlab 9.3 (R2017b). The T1 image of one subject from the UK study site was not available; the analysis is therefore based on the remaining 164 subjects. Anatomical T1 images were segmented into tissue classes using SPM’s new segment function. The resulting grey matter probability maps were normalized to the MNI space using the DARTEL toolbox ( Ashburner, 2007) persevering amount and smoothing maps with an 6mm isotropic full-width-half maximum (FWHM) Gaussian kernel. For the statistical comparison chronic back pain groups and healthy controls were compared employing an analysis of variance using study site as grouping factor and the total intracranial volume (calculated as the sum of the grey-matter, white-matter and CSF tissue classes) as a covariate for all subjects. Results were considered significant at p<0.05, whole-brain corrected for multiple comparisons using the FWE rate. However, we found no differences surviving correction for multiple comparisons across the full cohort (JP+UK+US), although some medial prefrontal cortex (adjacent to the pgACC) differences were found when limiting this analysis to UK patients only when exploring the data (see Supplementary File 1 for details).

An earlier version of this article can be found on bioRxiv ( https://doi.org/10.1101/223446).

## Results

### Classification using Machine Learning (Support Vector Machine)

Using *validation model 1* (i.e. training on the UK and Japan data and validating on the US data) and correlation as features for classification, the SVM framework correctly predicted 70%, p-value < 10 ^–3^, of patients (sensitivity) and 56%, p-value < 10 ^–3^, of controls (specificity), corresponding to a total accuracy of 63%, p-value < 10 ^–3^. Using *validation model 2* (i.e. training and testing using all available data, UK, Japan and US, with LTSO-CV) and the same features, the SVM framework correctly predicted 68%, p-value < 10 ^–3^, of patients (sensitivity) and 67%, p-value < 10 ^–3^ of controls (specificity), corresponding to a total accuracy of 68%, p-value < 10 ^–3^. The classification results are summarised in Table 2. Details of the top ten positive and negative weights are listed in Table 3 and Table 4.

**Table 2.** Support Vector Machine (SVM) classification results, showing the accuracy, sensitivity and specificity for the two validation models for pain, and also for gender and depression.

SVM classifier results (measure, p-value)			
Labels	Measure	Valid. model 1 UK+JP > US	Valid. model 2 LTSO-CV
Pain	Accuracy	**63** % (<10 ^-3^)	**68** % (<10 ^-3^)
	Sensitivity	**70** % (<10 ^-3^)	**68** % (<10 ^-3^)
	Specificity	**56** % (<10 ^-3^)	**67** % (<10 ^-3^)
Gender	Accuracy	48 % (1.00)	-
	Sensitivity	55 % (0.01)	-
	Specificity	41 % (1.00)	-
Depression	Accuracy	**59** % (<10 ^-3^)	-
	Sensitivity	**68** % (<10 ^-3^)	-
	Specificity	50 % (0.40)	-
BDI	Correlation	0.22 (0.07)	-

**Table 3.** Classification using Support Vector Machine. Top 10 positive weights based on validation model 1 (from UK, Japan data, tested on the US data).

Weight	ROI	ROI Name	MNI Centroid Coords
0.0207	83 – 128	R. olfactory sulc. — L. Hippocampus	(12,22,-18) – (-25,-22,-15)
0.0185	62 – 72	R orb. front. sulc. – L. ant. occipito-temporal lat. sulc.	(42,52,0) – (-41,-21,-28)
0.0179	42 – 111	R. cent. sulc. – R. post. inf. temp. sulc.	(42,-17,49) – (54,-60,1)
0.0178	51 – 128	L. ant. inf. frontal sulc. – L. Hippocampus	(-48,39,1) – (-25,-22,-15)
0.0174	81 – 138	R. occipito-polar sulc. – L. cerebellum	(15,-94,-4) – (-25,-61,-35)
0.0164	118 – 132	L. post. branch of sup. temporal sulc. – R. Caud.	(-47,-66,17) – (13,10,10)
0.0163	109 – 132	R. ant. infer. temp. sul. – R. Caudate	(62,-24,-19) – (13,10,10)
0.0162	13 – 111	L. ant. sub-cent. ramus lat. fiss. – R. post. inf. temp. sulc.	(-48,0,7) – (54,-60,1)
0.0155	43 – 111	L. cent. sylvian sulc. – R. post. inf. temp. sulc.	(-60,-2,16) – (54,-60,1)
0.0139	61 – 66	L. orb. front. sulc. – R. sup. frontal sulc.	(-41,50,1) – (26,24,49)

**Table 4.** Classification using support vector machine. Top 10 negative weights based on validation model 1 (from UK, Japan data, tested on the US data).

Weight	ROI	ROI Name	MNI Centroid Coords
-0.0192	69 – 135	R. ant. intralingual sulc. – R. Hippocampus	(15,-60,-4) – (27,-20,-15)
-0.0168	16 – 62	R. post. sub-cent. ramus lat. fissure – R. orb. frontal sulc.	(49,-12,16) – (42,52,0)
-0.0164	8 – 87	R. asc. ramus of lat. fissure – R. int. parietal sulc.	(50,19,5) – (6,-56,44)
-0.0162	26 – 61	R. sup. postcent. intraparietal sulc. – L. orb. frontal sulc.	(48,-28,48) – (-41,50,1)
-0.0157	106 – 117	L. rhinal sulc. – R. ant. branch of sup. temporal sulc.	(-26,-7,-36) – (56,-47,27)
-0.0154	62 – 124	R. orbital frontal sulc. – L. Thalamus	(42,52,0) – (-10,-19,7)
-0.0152	1 – 100	L. ant. lateral fissure — L. sup. precentral sulc.	(-33,13,-21) – (-39,-6,51)
-0.014	20 – 21	R. calloso-marginal post. fissure – Left calcarine fissure	(8,-27,45) – (-10,-65,4)
-0.0143	6 – 62	R. ant. ramus of lat. fissure – R. orbital frontal sulc.	(45,27,-2) – (42,52,0)
-0.0140	26 – 42	R. sup. postcentral intraparietal sulc. – R. central sulc.	(48,-28,48) – (42,-17,49)

To test whether these results were driven by confounds such as gender (the sample was not perfectly gender balanced) or depression (many patients also had a depression diagnosis) we tested two new models where instead of the original labels (patient or control) we used ‘male’ and ‘female’ and ‘depressed’ and ‘not-depressed’, respectively. We used exactly the same SVM framework described above and *validation model 1* (trained on UK and Japan data and validated on US data). To obtain the depression-related labels we divided the subjects according to their Beck Depression Inventory (BDI) scores: BDI ≥ 3 (depressed), BDI < 3 (not-depressed). This was done to generate a roughly equal division of the data set into two groups, with ‘low’ and ‘high’ BDI scores, accepting the fact that this value has no particular clinical significance. Using a higher value (BDI=10) produced unequal groups and although the classifier did not produce above-chance classification on this basis, this is difficult to interpret given the limited power. Note that there was no correlation between pain VAS and BDI scores.

The accuracy of the gender model was only 48% (p-value = 1.00) with sensitivity = 55% (p-value = 0.01) and specificity = 41% (p-value = 1.00). The accuracy of the depression model, although statistically significant, was lower than with the pain-related labels: accuracy = 59% (p-value < 10 ^–3^), sensitivity = 68% (p-value < 10 ^–3^), specificity = 50% (p-value = 0.40). This result was expected given that the depression labels are highly correlated with the pain labels.

Finally, we also tested if the output of the classifier for each validation sample (i.e. how far is the sample from the decision boundary for both sides) correlated with the BDI score for each individual. The correlation was found to be low (0.22) and not statistically significant (p-value = 0.074). This result together with the two confound models are consistent with the hypothesis that the classifier is primarily related to pain.

### Classification using Deep Neural Networks

Deep Learning algorithms represent a novel approach to classification for complex data sets, and have recently been applied to neuroimaging data ( Kim *et al.*, 2016; Plis *et al.*, 2014; Suk & Shen, 2013). Here, we used a CVAE with the units of *n*_1_ = 100, *n*_2_ = 50, and *n*_3_ = 10, which achieved the best validation accuracy for *validation model 1* (i.e. training on the UK and Japan data and validating on the US data). The CVAE correctly predicted 55% of patients (sensitivity) and 72% of controls (specificity) on average of 100 trials, corresponding to a total accuracy of 63%. Recall that we built a model *p _k_*( *X*| *y*) for *k*-th trial. We used the likelihoods ∏ _*k*_ *p _k_*( *X*| *y*) of all the 100 models for an ensemble. Then, ∏ _*k*_ *p _k_*( *X*| *y* = 1) *p*( *y* = 1) > ∏ _*k*_ *p _k_*( *X*| *y* = 0) *p*( *y* = 0) indicates that the subject is classified into the class *y* = 1. The ensemble achieved a total accuracy of 68%. Contribution weights varied over trials, and were relatively evenly matched across contributing nodes: Table 5 summarizes the regions which were frequently highly weighted.

**Table 5.** Classification with deep learning. ROIs which are frequently significant in the CVAE for validation 1 with networks D=default; CO=cingulo-opercular; S=sensorimotor.

Frequency	ROI	ROI name	Network	MNI coord.
0.116	41	Left central sulcus	S	(-41,-20,48)
0.116	36	Right insula	S	(42,4,2)
0.116	83	Right olfactory sulcus	D	(12,22,-18)
0.114	59	Left median frontal sulcus	D	(-15,20,58)
0.112	44	Right central sylvian sulcus	S	(61,0,17)
0.111	23	Left collateral fissure	D	(-25,-45,-13)
0.111	46	Right subcallosal sulcus	D	(4,-14,25)
0.111	40	Right paracentral lobule central sulcus	CO	(4,-30,55)
0.110	33	Left parieto-occipital fissure	D	(-9,-69,22)
0.110	42	Right central sulcus	S	(42,-17,49)

Using *validation model 2* (i.e. pooling the three datasets together (UK, Japan and US) and using an LTSO CV), the CVAE correctly predicted 56% of patients (sensitivity) and 71% of controls (specificity) on average of 10 trials, corresponding to a total accuracy of 64%. The ensemble of the 10 trials achieved a total accuracy of 68%.

### Characterisation of network changes: Hub Disruption

**Figure 2.**
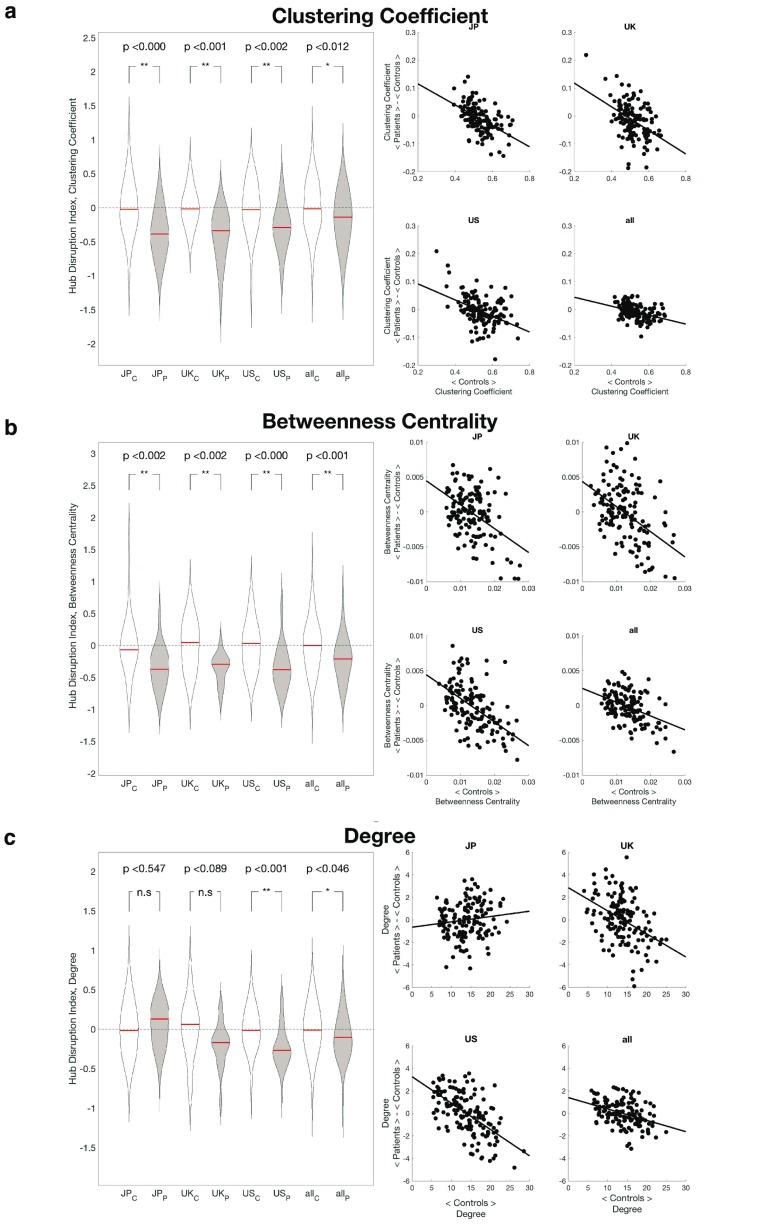
Hub disruption results for **a**) Clustering coefficient, **b**) Betweenness centrality, and **c**) Degree. The figure shows the HDI index individually for each site, and for the entire dataset. For each metric, we show the distribution of subject-wise HDI on the left panels, and the scatter plot of the ROI-specific changes in nodal graph metric on the right panels.

Evidence of reliable network-based classification indicates a possible disturbance of network topology in chronic pain. One way to investigate this further is to apply graph theoretic measures, which allow characteristation of the basic network topology of brain networks ( Rubinov & Sporns, 2010). This approach has been widely applied to brain data across a range of psychiatric and neurological conditions ( Bressler & Menon, 2010; Bullmore & Sporns, 2009). Of particular relevance is ’hub disruption’, which refers to a change in the nodal graph topology for any individual metric across the whole brain ( Achard *et al.*, 2012). It has previously been shown that brain networks undergo hub disruption for *degree* (the number of connections for each node) in chronic pain, with evidence from both in human chronic back pain patients and rodent pain models ( Mansour *et al.*, 2016). Here, we estimated Hub Disruption indices across all 3 data sets using a range of nodal graph metrics (see methods). As shown in Figure 2, we found changes in HDI for clustering coefficient and betweenness centrality consistently across all 3 data sets, and evidence for changes in degree HDI in the US cohort, but not other cohorts.

### Characterisation of network changes: Modular Reorganisation

The modular structure of the brain - the fact that certain groups of brain regions are especially well-connected with each other, is one of the fundamental properties of brain networks ( Meunier *et al.*, 2010; Nicolini & Bifone, 2016; Sporns & Betzel, 2016). Different modules reflect information processing subnetworks that have some degree of independence from each other. The pattern of changes in hub disruption index might suggest a change in the underlying modularity of the network. More specifically, a reduction in the extent to which nodes tend to cluster together, and a reduction in betweenness centrality (the number of shortest paths between nodes that pass through a node), in the absence of other aspects of hub disruption, could relate to a reorganisation of the modular architecture of the network.

To investigate the pattern of modular reorganisation across our chronic pain and control datasets, we developed a method to estimate the common modular architecture across all subjects in each group. Specifically, we computed the multislice modularity within each group, which effectively couples together all subjects within each group into a single large network, and estimates the modular structure of this graph to compute a *consensus* (or ‘ *agreement*’) matrix ( Lancichinetti & Fortunato, 2012; Mucha *et al.*, 2010). Then, we computed the difference between the agreement matrix for the chronic pain and control groups, to determine the agreement difference matrix ( Figure 1). This matrix consists of positive (red) and negative (blue) values. The positive values reflect pairs of nodes that are estimated to appear more commonly in the same module in pain patients, and the negative values represent pairs of nodes that are estimated to appear less commonly in pain patients. We defined the overall modular reorganisation for each node as the sum of both positive and negative values for each node (i.e. the sum of each column in the agreement difference matrix). That is, the larger the value, the greater the reorganisation (purple plot in Figure 1).

**Figure 3.**
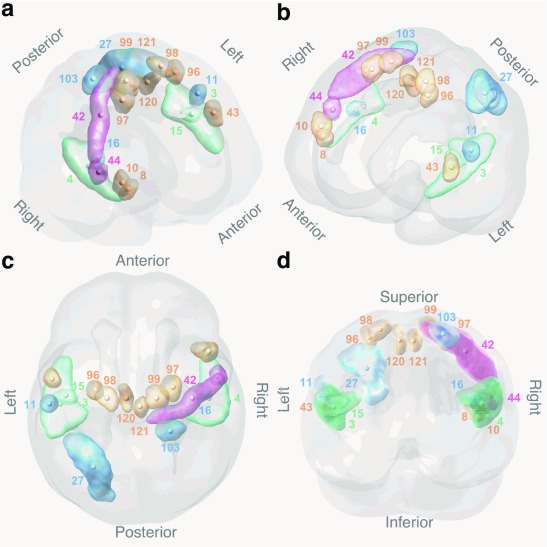
Brain regions showing modular reorganisation. Anterior view from top left ( **a**) and top right ( **b**), superior view ( **c**), and posterior view ( **d**) show 19 brain ROIs with the best evidence for modular reorganisation in the pain group, compared to the control group, based on the arbitrary threshold of *p* < 0.01, as listed in Table 6. The ROIs are colour coded according to their basic anatomical region (cortical lobe): ROIs in frontal lobe in light orange, frontoparietal lobe in light magenta, parietal lobe in light blue, and temperoparietal lobe in light green.

To statistically evaluate the values, we performed a permutation test of sum reorganisation estimation, to yield one-sided *p*-values across all ROIs. As illustrated in Figure 3 and Table 6 (at a threshold of *p* < 0.01), changes were seen across widespread bilateral sensorimotor cortical regions. We also saw significant changes in right inferolateral prefrontal cortex, bilateral temporal cortical regions, and left intraparietal sulcus.

**Table 6.** Brain ROIs that show modular reorganisation at a cut-off threshold of *p* < 0.01. The table lists the ROI by number (in the BSA-AAL composite atlas), with its corresponding anatomical label, region and MNI coordinates. The overall modularity reorganisation metric *AD* is listed, included it’s decomposition into positive and negative contributory factors. For anatomy, F=frontal; TP=temporoparietal; FP=frontoparietal; P=parietal.

*AD* (+/-)	p-value	ROI	Anatomical label	Anat.	MNI coord.
10.63 (4.23, -6.39)	0.001	8	R. ascending ramus of the lat. fissure	F	(50,19,5)
9.20 (3.54, -5.66)	0.001	10	R. diagonal ramus of the lat. fissure	F	(54,17,12)
14.30 (5.29, -9.01)	0.002	3	L. post. lat. fissure	TP	(-54,-20,10)
12.84 (4.62, -8.22)	0.002	16	R. post. sub-cent. ramus of the lat. fissure	P	(49,-12,16)
12.02 (5.59, -6.43)	0.002	27	L. intraparietal sulcus	P	(-30,-66,40)
8.20 (1.52, -6.68)	0.003	98	L. median precentral sulcus	F	(-20,-15,66)
11.10 (3.67, -7.42)	0.004	11	L. retrocent. trans. ramus of lat. fissure	P	(-62,-20,23)
12.82 (4.59, -8.24)	0.004	15	L. post. sub-central ramus of the lat. fissure	TP	(-50,-15,13)
8.50 (1.68, -6.83)	0.004	39	L. paracentral lobule central sulcus	FP	(-5,-29,57)
11.61 (3.90, -7.71)	0.004	43	L. central sylvian sulcus	F	(-60,-2,16)
13.88 (5.12, -8.76)	0.005	4	R. post. lat. fissure	TP	(55,-15,13)
7.67 (1.27, -6.40)	0.006	42	R. central sulcus	FP	(42,-17,49)
7.84 (1.35, -6.49)	0.006	99	R. median precentral sulcus	F	(17,-15,68)
8.15 (1.45, -6.70)	0.006	103	R. sup. postcentral sulcus	P	(26,-39,63)
7.78 (1.34, -6.39)	0.006	120	L. paracentral sulcus	F	(-6,-16,58)
10.48 (3.20, -7.28)	0.007	44	R. central sylvian sulcus	FP	(61,0,17)
8.17 (1.46, -6.72)	0.007	96	L. marginal precentral sulcus	F	(-28,-11,60)
8.52 (1.68, -6.84)	0.007	97	R. marginal precentral sulcus	F	(27,-8,61)
8.04 (1.43, -6.62)	0.007	121	R. paracentral sulcus	F	(5,-22,58)

The modular reorganisation analysis allows us to consider separately positive and negative reorganisation values (see also Table 7). In sensorimotor cortex, we observed that reorganisation tended to be dominated by negative values i.e. reflecting a reduction in the tendency of these regions to form modular ’partners’ in chronic pain. This was in fact the most common pattern across most brain regions (i.e. in Table 6), suggesting that negative modular reorganisation reflects the broad characteristic feature of chronic pain. The region with the highest positive reorganisation was the left intraparietal sulcus. Indeed this was the only region identified in our overall sum modular reorganisation at a discovery threshold of p<0.01 ( Table 6) that had a reasonable significance level (p<0.014) when restricting the analysis to purely positive reorganisation ( Table 7).

**Table 7.** Modular brain reorganisation at cut-off threshold of *p* < 0.05 considering only positive or negative agreement difference values *AD*. The table lists the ROI by number (in the BSA-AAL composite atlas), with its corresponding anatomical label, region and MNI coordinates. Networks are D=default; O=occipital.

*AD* (pos.)	p-value	ROI	Anatomical label	Network	MNI coord.
5.59	0.014	27	Left intraparietal sulcus	D	(-30,-66,40)
3.75	0.041	75	Right internal occipito-temporal lateral	O	(36,-56,-17)
5.73	0.043	76	sulcus Left median occipito-temporal lateral sulcus	O	(-48,-48,-20)
*AD* (neg.)	p-value	ROI	Anatomical label	Network	MNI coord.
-0.84	0.027	70	Left posterior intra-lingual sulcus	O	(-6,-76,-9)
-0.90	0.030	69	Right anterior intralingual sulcus	O	(15,-60,-4)
-0.90	0.033	71	Right posterior intra-lingual sulcus	O	(10,-73,-6)
-0.96	0.048	47	Left cuneal sulcus	O	(-4,-86,17)

**Figure 4.**
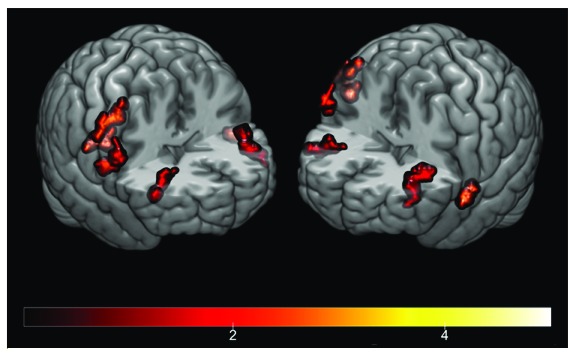
Brain regions showing increased connectivity with bilateral pgACC seeds in pain > controls. This identifies bilateral regions of sensorimotor cortex, including premotor and lateral prefrontal regions (See Table 8 for coordinates and statistics).

Finally, since recent research has highlighted a potentially important role for the pregenual anterior cingulate cortex (pgACC) in endogenous control of persistent pain, which would provide a mechanistic link to psychological theories of pain which highlight resilience and fear-avoidance. We therefore tested whether pgACC connectivity was different in the patient group (across all data sets). We found pgACC showed enhanced connectivity with regions of sensorimotor cortex, including several areas overlapping those identified in our network modularity reorganization analysis ( Figure 4, Table 8).

**Table 8.** Brain regions associated with increased pgACC seed connectivity in chronic pain versus controls. The table lists regions showing cluster-level FWE corrected significant regions in bilateral sensorimotor/premotor and lateral prefrontal regions (see Figure 4).

Coords (x,y,z)	Peak-level FWE-corr	Cluster-level FWE-corr	t	equivZ
58,12,32	0.214	0.003	4.98	4.79.
-36,38,14	0.359	0.001	4.82.	4.65
58,-4,46	0.604	0.005	4.62	4.46
-58,8,-4	0.800	0.006	4.46	4.32
52,-4,32	0.966	0.014	4.24	4.12
48,40,10	0.966	0.002	4.24	4.12

## Discussion

The results show that there are sufficient brain network changes in chronic back pain to allow reliable classification. Furthermore, the way in which networks are changed follows a characteristic pattern, with global disruption of hub connectivity and modular reorganisation. In particular, we show that bilateral sensorimotor cortical regions undergo the substantial reorganisation, including in regions that also carry predictive weight in classification.

Since chronic pain dominates many aspects of cognition and action, the existence of widespread connectivity changes is not unexpected ( Baliki *et al.*, 2011; Baliki *et al.*, 2008; Hashmi *et al.*, 2013; Hemington *et al.*, 2016; Kutch *et al.*, 2017; Napadow *et al.*, 2010; Tagliazucchi *et al.*, 2010). A challenge, therefore, is to try and identify regions that may have an important or driving role in pain. The approach we take here looks across several methods: connectivity-based machine learning to identify changes important for classification, and a modularity analysis to identify brain regions that show fundamental changes in their functional network identity. Although it is not possible to differentiate causal from consequential connectivity changes, these methods can identify regions that appear to be important in chronic pain at an informational level.

In particular, we present a network modularity analysis approach that aims to identify brain regions that are reorganised in chronic pain. Modular reorganisation is defined on the basis of connections from a particular ROI that appear to join or leave modules with other ROIs - effectively reflecting a change in the ROIs modular identity. This analysis identifies a number of brain regions, but was clearly dominated by sensorimotor cortical regions (i.e. sensory, motor, and premotor cortex). Sensorimotor cortex has been consistently implicated in chronic pain ( Eto *et al.*, 2011; Flor *et al.*, 1997; Kim & Nabekura, 2011; Kuner & Flor, 2017; Kutch *et al.*, 2017; Yanagisawa *et al.*, 2016), and consequently is a well-recognised target for direct intervention (for instance by stimulation or neurofeedback). The efficacy of these interventions implies an important role of sensorimotor cortex in chronic pain experience ( Antal *et al.*, 2010; Garcia-Larrea *et al.*, 1999; Hosomi *et al.*, 2008; Tsubokawa *et al.*, 1991; Yanagisawa *et al.*, 2016). In machine learning studies, voxels in SI have been shown to carry the greatest weight in classifiers trained on BOLD responses during experimental electrical lower back stimulation ( Callan *et al.*, 2014), and structural image decoding analyses identifies high classification weights in sensory and motor cortices (amongst other regions) ( Koush *et al.*, 2013; Ung *et al.*, 2012). Overall, this is consistent with an important role for multiple subregions of sensorimotor cortex in the chronic back pain state. But notably, however, we find less evidence of striatal-prefrontal regions, and cingulo-opercular regions in our analyses. Although these regions have a demonstrated role in chronic pain, the evidence here does not support a fundamental reorganisation of them at a network level.

A particularly interesting finding in our data is that (left) intraparietal cortex displays a pattern of significant network change that is characterised by a relatively large amount of positive modular reorganisation. That is, it appears to enhance its modular links with other brain regions, hinting at a potentially important role in generating the chronic pain state. As an unexpected finding it should be interpreted cautiously, but it could relate to the regions involvement in perceptual-motor coordination and ( Grefkes & Fink, 2005) or multisensory peripersonal attention ( Makin *et al.*, 2007) - a hypothesis that would benefit from further study.

The observation that pgACC - sensorimotor cortex is enhanced in chronic pain offers a potential link between networks involved in sensorimotor reorganisation and those involved in motivational and affective processing. Notably, the pgACC is a key node in the pain modulatory network - widely connected to cortical regions associated with pain and reward value and decision-making, and critically connected to the descending control system. It has been proposed to modulate pain based on the amount of prospective learnable information that pain onset or offset carries, based on computational estimates uncertainty ( Zhang *et al*., 2018). Furthermore, pgACC has been directly linked to chronic pain: for instance it lies close to medial PFC regions link to risk of developing chronic back pain ( Baliki *et al.*, 2012), and enhanced connectivity with PAG is seen in chronic neuropathic pain in a symptom-specific manner ( Segerdahl *et al*., 2018)

Network changes may arise in chronic pain for a variety of distinct reasons, and it is difficult to distinguish these on the basis of an rsfMRI scan at a single point in time. For instance, some regions may have a primary causative or risk factor role in the clinical manifestation of chronic pain, and therefore might be expected to be apparent before chronic pain is itself established (striatal - medial prefrontal cortical regions are candidate regions for this ( Baliki *et al.*, 2012)). Alternatively, other regions might have no role in the cause or expression of chronic pain, but instead reflect consequential (i.e. downstream) changes, for example perceptual learning of a new sensory environment in which pain is more common ( Mancini *et al.*, 2016; Mano *et al.*, 2017). Such regions might be expected to manifest later, and resolve with successful pain treatment ( Rodriguez-Raecke *et al.*, 2009).

Further complexities of studying network changes in pain relate to confounding factors such as medication use, secondary effects of pain such as disability, and co-morbid disease such as depression. We also cannot determine the specificity of our results to chronic back pain, as opposed to other chronic pain conditions, and recent evidence suggests that many aspects of network changes may be common ( Baliki *et al.*, 2014; Mansour *et al.*, 2016). Hence future network studies would be greatly enhanced by longitudinal data (and pre-morbid data when available), better identifying correlations with pain severity, evaluation of response to drugs, and use of open data sources to provide larger data sets to test generalisation across diagnoses. This should allow distinction of components of the network that reflect the cumulative impact of chronic pain, from those reflect a state-dependent biomarker for ongoing symptomatic experience.

Methodological caveats that should be noted are that identification of network changes may depend on the brain atlas used. Higher resolution atlas (i.e. greater number of smaller ROIs) may have a better ability to detection small regions that are important, but greatly increase the numbers of features for classification, which can lead to spurious over-fitting and worse generalisation of the results.

In terms of classification methods, the accuracy of the SVM is comparable with that seen in other machine learning-based disease biomarkers that have used independent validation cohorts ( Takagi *et al.*, 2017; Yahata *et al.*, 2016) (albeit less than that seen with classifiers for phasic BOLD responses to acute painful stimulation in healthy individuals ( Wager *et al.*, 2013)). Here, we also applied a deep learning approach using deep convolutional neural networks, the utility of which has not previously been tested in chronic pain. Deep networks are best known for solving natural image recognition problems with high accuracy, often using very large training data sets. This is typically necessary since they have to extract features from images automatically. With smaller data sets, accuracy is reduced, but performance may still be strong, and this has led to their application to human neuroimaging data. Here, we used a CVAE with a small number of network layers, which suppresses over-fitting in return for a lower classification accuracy than ordinary deep neural networks. An important difference between the connectivity-based decoding and the deep neural network is that the input to the latter is the ROI time-series, not a correlation matrix. In principle, this allows it to use nonlinear and non-pairwise correlations between ROIs implicitly, and hence confers the capacity for much more complex feature extraction. This means that performance may improve when new data becomes available, and help to make deep neural networks a promising method for future classifiers and biomarkers.

In terms of theories of chronic pain, the data here support the general notion of chronic pain as a network disorder, albeit with different aspects of specific regions of the network disturbed in different ways. This approach adds to and complement a substantial body of studies identifying and characterising network changes in chronic pain ( Apkarian *et al.*, 2009). The limitations of rsfMRI network analysis also emphasises the importance of understanding the underlying behaviour and computational function of network nodes in chronic pain (for instance, the intraparietal sulcus), and data-driven methods should ideally complement hypothesis-driven task-based studies in clinical groups. Notwithstanding this, a particularly attractive property of the network-theory based approach is their translational applicability to animal models, since topological metrics are relatively independent of brain morphology. In principle, this allows targeted experimental interventions to test whether there is a direct relationship between network specific changes and the manifestation of chronic pain.

## Data availability

Pre-processed fMRI data from both UK and Japan are available at the ATR Open Access Database (as raw connectivity matrices). Application form and license information can be found here. Data is available by emailing decnef-db-admin@atr.jp.

Raw data are available from the OpenPain Project (OPP), which is where we sourced the validation fMRI data (Principal Investigator: A. Vania Apkarian). Licence information is found here, and access to data is provided by registering here.

SVM data analysis code for fMRI data (PRoNTO) was co-written by MR and is available at http://www.mlnl.cs.ucl.ac.uk/pronto/. The code for modularity reorganisation is available at: http://doi.org/10.5281/zenodo.1183399 ( leiken, 2018). Toolboxes for deep learning analyses are available at https://www.tensorflow.org/about/bib.

## Supplementary material

**Supplementary File 1.** This includes additional analysis and discussion related to issues raised by the reviewers.
