# Supplementary material for: Classification and characterisation of brain network changes in chronic back pain: A multicenter study
Source: Wellcome Open Res. 2018 Oct 10;3:19. Originally published 2018 Mar 1. [Version 2] doi: 10.12688/wellcomeopenres.14069.2 (PMC5930551; doi:10.12688/wellcomeopenres.14069.2)
Supplement: Supplementary file 1 [file wellcomeopenres-3-16193-s0000.tgz › 53fa7bdb-76b2-425d-b3a6-d17858e50d8d_Supplementary_File_1.docx]

The supplementary material below deals with specific issues raised in the review process:

**Relationship between pain and depression.**

The reviewers point out the fact that the use of a BDI of 3 has little or no relation to accepted thresholds for depression. The rationale for using BDI=3 was to generate an approximately 50% split of the patients into two groups, as an optimally-powered approach to see if the classifier was sensitive to BDI. However, we appreciate from a clinical perspective this value is arbitrary, and have now repeated the classification with a higher value (BDI=13, as suggested). This revealed chance level accuracy, although is not well especially well-powered because of the very unequal group sizes (accuracy: 0. 46; Sensitivity: 0. 86; Specificity: 0.13).

We have also computed the correlation between pain and depression, which revealed no significant correlations:

| Site | Cohort | r | p |
| --- | --- | --- | --- |
| JP | Controls | 0.099 | 0.556 |
|  | Patients | 0.394 | 0.077 |
| UK | Controls | 0.198 | 0.447 |
|  | Patients | 0.199 | 0.443 |

We have updated the manuscript (Results section) as follows:

‘This was done to generate a roughly equal division of the data set into two groups, with ‘low’ and ‘high’ BDI scores, accepting the fact that this value has no particular clinical significance. Using a higher value (BDI=10) produced unequal groups and although the classifier did not produce above-chance classification on this basis, this is difficult to interpret given the limited power. Note that there was no correlation between pain VAS and BDI scores.’

Another point raised was whether the classification could be corrected for BDI score with respect to pain. In our analysis, we simply restricted ourselves to a binary discrimination, and so it is not clear how we can do this. However, we would agree that a future step would be to take a  multivariate regression approach (e.g. using Gaussian Processes, Marquand, et al. *Neuroimage* 49.3 (2010): 2178-2189.), whereby this could be done.

**Hub disruption**

The authors raise the question of whether the HDI analysis is robust to random removal of links. In general the answer is yes. Below we detail the analyses performed:

1. Removal of top 5% of nodes for our three primary graph measures (in All subjects - Japan, UK and USA). The graphs show the correlation between the the HDI before (K) and after (K’) removal of the top nodes.


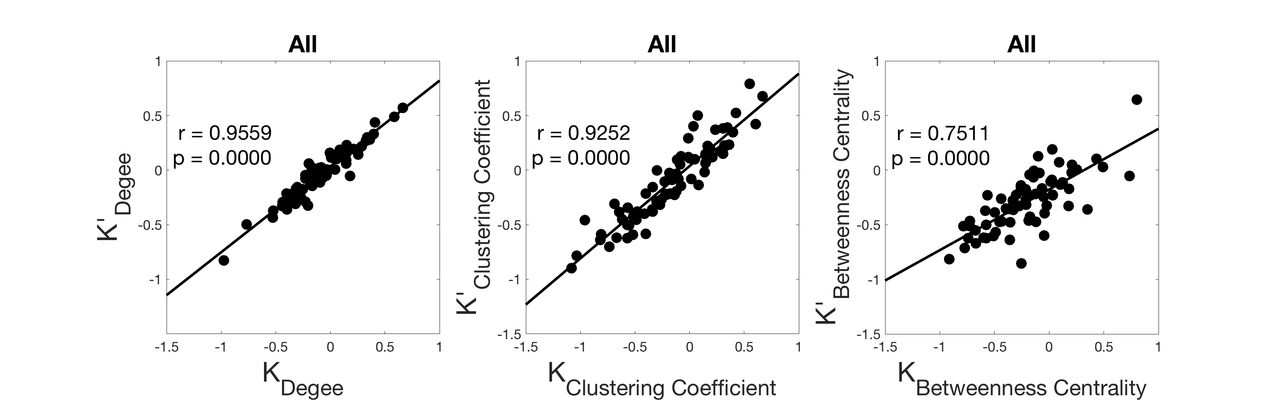


Figure R1. Correlation between K and K' (after a removal of rank order top 5% nodes) for **Left**) Degree, **Middle**) Clustering Coefficient, and **Right**) Betweenness centrality for the entire dataset (All: JP+UK+US).

1. Random removal of 90% of nodes. Here, the analysis is repeated multiple times with different random node removals, and the aggregate and mean presented in the graph below.


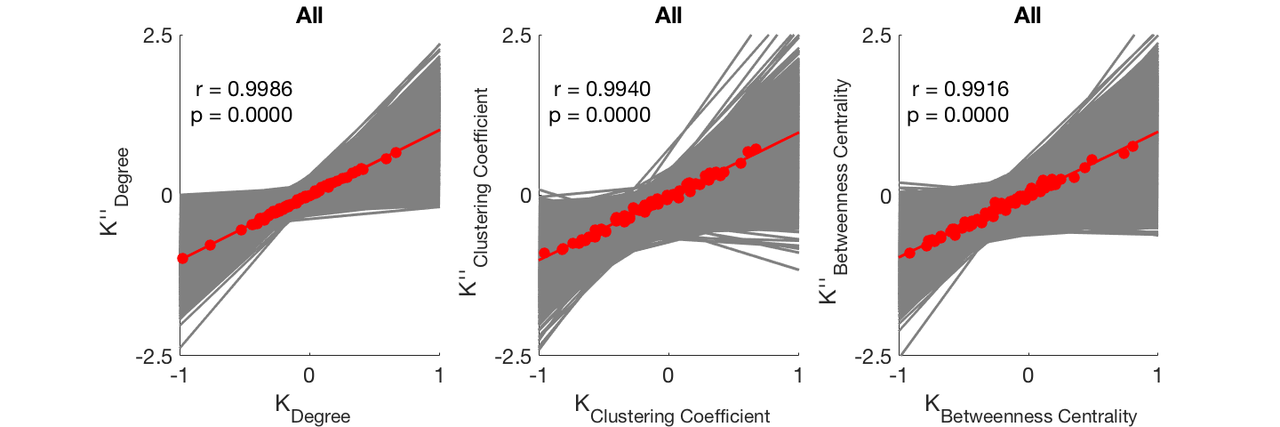


Figure R2. Correlation between K and K’’ (after a random removal of 90% nodes, i.e. from a random 10% subset of nodes) for **Left**) Degree, **Middle**) Clustering Coefficient, and **Right**) Betweenness centrality for the entire dataset(All: JP+UK+US). Across 5000 iterations with a random removal of 90% nodes, the correlation between K and K'' varied each time to some extent, presumably due to a small number of the total number of ROI. The shades and the slopes in gray depict an empirical 95% confident interval derived from the iterations, and the slope and the points in red depict an average over the iterations.

We now add the following text to the manuscript (methods section) to direct the reader to these analyses:

“In response to reviewers comments, we also considered if the HDI was robust to either removal of the top 5% of nodes, or random removal of 90% of nodes, which it was (see rebuttal letter for further details).”

A further question was raised about the use of hub metrics for classification. In our analysis as it stands, we can’t directly use the HDI for classification, since this suffers from an information leak problem: that is, the calculation of HDI is based on the average values from the control groups. For the HDI to be truly independent, we would need an independent control group to provide ‘normative values’ against which both our patient and control groups could be evaluated (such a sample is not yet available to us). It is worth mentioning that the HDI itself is a relatively non-specific measure of brain network changes, and the way it is calculated renders it susceptible to data noise (which will yield a significantly non-zero HDI). Therefore it is important that normal values be calculated under identical conditions (i.e. within scanner) if this is to be a viable method for future classification.

In the Methods section, we now write:

‘It should be noted that the way HDI is calculated makes it intrinsically susceptible to data noise, which will tend to produce significant values. In addition, because the control group are used to define the normal values against which the patients are compared, we cannot use the individual values for classification, since the values are not independent (i.e. there is ‘information leak’ between the classification sets).’

We did directly use global graph metrics calculated in individual subjects as features in classification i.e. as a method of dimensionality reduction, but this performed less well than simply using the connectivity matrices. However, it is still possible that an appropriately specified subset of nodal graph metrics could perform well as classification features in future studies.

**SVM analysis**

Following a similar logic, as suggested by the reviewers, we also tested the robustness of the SVM classifier with removal top links. To do this, we removed the top 10 +ve and top 10 -ve links as defined by the connectivity correlation coefficient. This decreased the classification accuracy by 2.0%, which we interpret as evidence that the classification is relatively robust.

The reviewers also ask whether the predicted chronic pain scores (e.g., distance from the SVM hyperplane) correlated with gender, depression, head movement, or other variables, and whether any correlation is actually greater than that with chronic pain. We performed a multiple linear regression of these parameters, which revealed no significant correlation

    Parameter Estimate        SE        tStat       pValue

                    _________    ________    ________    ________

    (Intercept)      -0.16578     0.68849    -0.24079     0.81185

    BDI              0.015683    0.019834     0.79075     0.43717

    PainDuration    -0.004412    0.010203    -0.43242     0.66947

    PainVAS          0.061735    0.069006     0.89463     0.38025

    Gender_f         -0.41834     0.20486     -2.0421    0.052773

    FD                  5.241      2.8535      1.8367    0.079209

    DVARS            -0.40055      0.5008    -0.79982       0.432

    MeanCorrCoef       21.777      16.697      1.3042     0.20505

**Functional connectivity**.

The reviewers ask several questions related to the specific identity of network connections implicated in chronic back pain, and how they might relate to known pathways involved in nociceptive / extra-nociceptive / descending modulation regions/functions, and to behavioural measures and other studies. We agree this is a very important issue, as it might allow translation of the findings to current understanding of pain processing networks in the brain. Indeed this may provide a critical link between data-driven analysis of whole brain networks, and hypothesis-driven analysis related to specific pain functions (a point we already argue for in the discussion).

With this in mind, our recent research has highlighted a potentially important role for the pregenual anterior cingulate cortex (pgACC) in endogenous control of persistent pain (Zhang et al, 2018, eLife). This effectively provides a mechanism by which short-term pain can be overcome whilst people actively seek to relieve it, and a deficiency with such a mechanism would offer a clear mechanistic link to psychological theories of pain - namely the fear-avoidance model. The pgACC also lies within the MPFC, which is directly implicated in back pain chronification (Baliki et al, 2012). We therefore tested whether pgACC connectivity was different in the patient group (across all data sets). We found that pgACC showed enhanced connectivity with regions of sensorimotor cortex, including several areas overlapping those identified in our network modularity reorganization analysis. This offers a potential link between sensorimotor reorganization and motivational / endogenous control functions.

We added the following text to the abstract, methods, results and discussion:

Abstract: Furthermore, these regions were found to display increased connectivity with the pregenual anterior cingulate cortex, a region known to be involved in endogenous pain control.

Methods: Pregenual ACC connectivity analysis.

This was based on the same preprocessing pipeline as above. Voxel-wise maps of connectivity, based on Fisher-transformed correlations of voxel-based BOLD time series, were computed to evaluate regions that were more or less correlated in patients than controls. This was based on a bilateral pgACC seed was a 6mm diameter sphere centered on [+/-3,40,5], based on our recent study identifying this region in endogenous control of persistent pain in healthy subjects (Zhang et al, 2018). Statistical analysis was based on simple t-contrasts.

Results: Finally, since recent research has highlighted a potentially important role for the pregenual anterior cingulate cortex (pgACC) in endogenous control of persistent pain, which would provide a mechanistic link to psychological theories of pain which highlight resilience and fear-avoidance. We therefore tested whether pgACC connectivity was different in the patient group (across all data sets). We found pgACC showed enhanced connectivity with regions of sensorimotor cortex, including several areas overlapping those identified in our network modularity reorganization analysis (fig 4, Table 8).


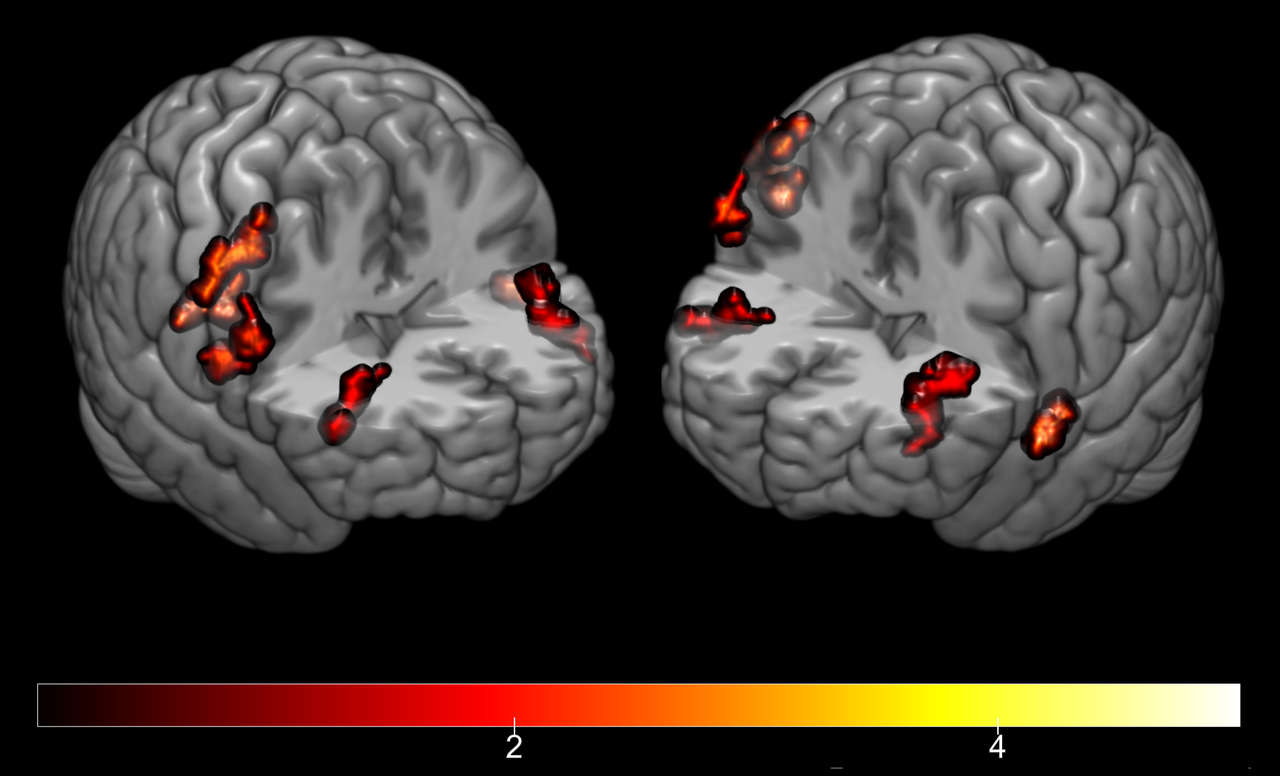


Figure 4: Brain regions showing increased connectivity with bilateral pgACC seeds in pain > controls. This identifies bilateral regions of sensorimotor cortex, including  premotor and lateral prefrontal regions (See Table 8 for coordinates and statistics)

Discussion. The observation that pgACC - sensorimotor cortex is enhanced in chronic pain offers a potential link between networks involved in sensorimotor reorganisation and those involved in motivational and affective processing. Notably, the pgACC is a key node in the pain modulatory network - widely connected to cortical regions associated with pain and reward value and decision-making, and critically connected to the descending control system. It has been proposed to modulate pain based on the amount of prospective learnable information that pain onset or offset carries, based on computational estimates uncertainty (Zhang et al, 2018). Furthermore, pgACC has been directly linked to chronic pain: for instance it lies close to medial PFC regions linked to risk of developing chronic back pain (Baliki et al, 2012), and enhanced connectivity with PAG is seen in chronic neuropathic pain in a symptom-specific manner (Segerdahl, 2018).

**Grey matter volume.**

The reviewers also asked whether GM volume differed between the two groups. We first tested for reduced grey matter volume in chronic back pain patients compared to healthy controls across all three study sites using a conjunction analysis (global null hypothesis). No significant results were observed. Likewise no significant results were found when testing for the reverse contrast. Subsequently, we compared grey matter volumes between groups separately for the three study sites. We found a significant lower grey matter volume in UK chronic back pain patients compared to controls in a region adjacent to the perigenual anterior cingulate cortex ([18,39,-6], z=5.2, p_FWE_=0.012). No further significant group differences were observed.

Figure:


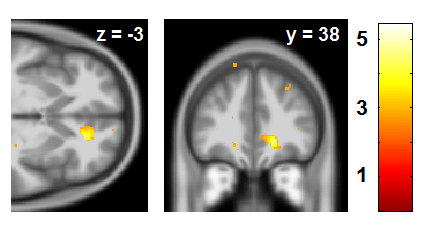


Visualization threshold is p<0.005.

We now add additional sections the manuscript, as follows

Methods: We also considered whether there were grey matter changes between groups. Data were analyzed with SPM12 and Matlab 9.3 (R2017b). The T1 image of one subject from the UK study site was not available; the analysis is therefore based on the remaining 164 subjects. Anatomical T1 images were segmented into tissue classes using SPM’s new segment function. The resulting grey matter probability maps were normalized to the MNI space using the DARTEL toolbox (Ashburner 2007) persevering amount and smoothing maps with an 6mm isotropic full-width-half maximum (FWHM) Gaussian kernel. For the statistical comparison chronic back pain groups and healthy controls were compared employing an analysis of variance using study site as grouping factor and the total intracranial volume (calculated as the sum of the grey-matter, white-matter and CSF tissue classes) as a covariate for all subjects. Results were considered significant at p<0.05, whole-brain corrected for multiple comparisons using the FWE rate. However, we found no differences surviving correction for multiple comparisons across the full cohort (JP+UK+US), although some medial prefrontal cortex (adjacent to the pgACC) differences were found when when limiting this analysis to UK patients only when exploring the data (see rebuttal letter for details).
