# Supplementary material for: Classification and characterisation of brain network changes in chronic back pain: A multicenter study
Source: Wellcome Open Res. 2018 Oct 10;3:19. Originally published 2018 Mar 1. [Version 2] doi: 10.12688/wellcomeopenres.14069.2 (PMC5930551; doi:10.12688/wellcomeopenres.14069.2)
Supplement: Peer review history

# Peer review: Referee response for version 1

**DOI:** 10.21956/wellcomeopenres.15303.r32965

**Published:** 2018-05-08

**Authors:** Jiro Kurata

**Affiliations:** 1 Department of Anesthesiology and Pain Clinic, Tokyo Medical and Dental University Hospital of Medicine, Tokyo, Japan

The present study by Mano et al. examined differences in brain network topology between 41 patients with chronic low back pain and 56 matched healthy controls, recruited at two sites in UK and Japan, using resting-state functional and structural MRI data. They performed an inter-regional correlation analysis over 140 predefined ROIs and classified the resultant connectivity matrices using Support Vector Machines into patient and control groups, and further validated the classifier on an independent dataset from the US (OpenPain Project) as well as on the total datasets. They described hub disruption over clustering coefficient, betweenness centrality, and degree; and highlighted brain modular reorganization at 7 specific areas including the left intraparietal sulcus in patients. They concluded feasibility of graph theory-based machine-learning classification in differentiating chronic pain patients and healthy controls.

 First, I would praise the authors' tremendous, sophisticated computational efforts in utilizing graph-theory analysis and machine-learning. Their findings on the specific network alterations and reorganized modules in chronic low back pain would potentially be good candidates for further inquiry into their pathophysiological significance, as they also stated in the conclusion remarks.

 So far they did not examine possible correlations between those graph theory-based and psychophysical parameters. Considering highly mathematical, theoretical nature of graph-theory analysis, it might rather be a good idea to reexamine more physiologically straightforward indices like functional connectivity and gray matter volume at those networks and modules that showed alteration in patients. A further correlation analysis with behavioral parameters would also be a plus.

 I was impressed by a dominant distribution of modular reorganization over the vast sensorimotor cortical areas. Those areas, as well as the intraparietal sulcus, might well be involved in plastic, functional and anatomical changes in chronic pain patients. As the authors mentioned, it might accord with many earlier neuroimaging studies as well as our recent unpublished data on functional connectivity and gray matter volume in those areas. I would be eager to see further results on possible associations between the current findings and the other imaging/behavioral parameters.

 Although hub disruption over the whole brain in patients might imply some global network reorganization or dysfunction, it remains unclear what kind of alterations in either pain perception, cognition, or other brain functions might be relevant to such phenomena. Could it be associated with, as it were, "well-being" of brain function as a whole? I would appreciate the authors' further analysis and comments on this significant finding.

 Overall, I would welcome and approve the present paper as it will add an indispensable piece of knowledge to cerebral pathophysiology of chronic pain.

I have read this submission. I believe that I have an appropriate level of expertise to confirm that it is of an acceptable scientific standard.

# Response

**Published:** 2018-09-27

**Authors:** Ben Seymour

**Affiliations:** Department of Engineering, University of Cambridge, UK

We thank the reviewers for their thoughtful and detailed evaluation. The new manuscript includes a supplement where we summarise the key issues that have been raised together by the 3 reviewers, and detail our response to them and the associated changes to the original manuscript that are in the revised version of the manuscript.

 We also want to highlight the valuable general commentary provided by the reviewers in relation to the broader aspects of network changes and biomarkers for chronic pain, and believe the open reviews offer a valuable perspective which greatly enhances the publication as a whole.

# Peer review: Referee response for version 1

**DOI:** 10.21956/wellcomeopenres.15303.r31330

**Published:** 2018-04-30

**Authors:** Tor D. Wager

**Affiliations:** 1 Department of Psychology and Neuroscience, University of Colorado Boulder, Boulder, CO, USA

This study investigates case-control differences between individuals with chronic back pain and matched controls across three independent cohorts. The authors take several complementary approaches to characterizing network-level changes in functional connectivity, including Support Vector Machine (SVM) classification of patients vs. controls, classification based on a deep neural network conditional variation auto encoder (CVAE), analysis of ‘hub disruption’, a global measure of functional network reorganization, and a modular reorganization analysis that assesses group-level changes in which regions belong to the same clusters (or ‘modules’) based on functional connectivity. Overall, the analyses are sophisticated and informative.

 One strength of the manuscript is that they assess the strength and of their results in terms of utility as a biomarker. Many studies show differences between patients and controls - but a key question unanswered by traditional analyses is how large those differences are, and whether they are related to back pain with sufficient effect sizes to allow correct classification at the individual-person level. Mano et al. take a prediction approach, demonstrating 70% sensitivity and 58% specificity for cases (6 months plus of back pain) vs. controls, when applied to a new cohort, and similar accuracy in another type of cross-validation. The CVAE performed similarly. This demonstrates that the results are replicable across studies and have at least limited utility as a biomarker.

 Embedded in these tests, and others, is another strength of the paper: the demonstration of generalizability. A recent trend in the literature is the sharing and pooling of data across sites. This allows for the aggregation of larger, more diverse samples, which is important for power, for the fidelity of the “picture” of brain changes with chronic pain and other disorders, and for establishing findings that generalize across laboratories and samples. This study is commendable in that it pools together data from 158 participants across three cohorts. In addition, the authors have already shared their data with the community. While multi-lab, multi-site analyses are becoming more common in some kinds of research - notably dementia, autism, and Parkinson’s - they are still very rare in chronic pain research. The MAPP pelvic pain consortium studies are a notable exception. This study demonstrates reproducibility across studies and a practice of data-sharing that will really help move the field forward. 

 A third strength of the study is the attempt to replicate and extend previous findings by Mansour et al. 2016, who found that chronic pain patients show signs of ‘hub disruption’ - changes in the hub status, broadly related to the number and strength of connections and a region’s role in brain networks - of brain regions across the brain. Mano et al. extend earlier results by testing several different hub metrics across the three datasets, and found the most consistent disruptions in clustering coefficient and betweenness centrality. It would be helpful here to also test how well these metrics work as a classifier, and what their correlation is with the severity and duration of chronic pain. As hub disruption does not provide any information about which brain regions and connections are driving the effects - which patterns because different groups may exhibit ‘hub disruption’ for different reasons, (e.g., it is related to loss of consciousness), it would also be helpful to further characterize the particular patterns underlying these global changes. Nonetheless, the convergence with previous findings, and generalization across studies, is promising.

 So what have we learned about the genesis and nature of chronic back pain? The study offers data on two, complementary perspectives. In the SVM analyses, some of the most important differences between patients and controls are found in fronto-striatal and fronto-hippocampal connections. (These are not discussed much in the paper, and it would be interesting to hear the authors’ perspective). This matches other recent studies in humans and rodents suggesting that fronto-hippocampal and fronto-striatal circuits play an important role in chronic pain. It would  be helpful to visualize  these findings in the paper and examine convergence with other analyses. The modular reorganization analyses paints a somewhat different picture: Sensorimotor reorganization, and in particular reduced tendency for sensorimotor regions to couple with other regions. Though the jury is still out, it is tempting to speculate that these changes are similar to those that might occur with evoked, nociceptive pain. However, comparable analyses have not yet been done for evoked pain, so it’s difficult to tell. 

 What is at stake is whether chronic back pain involves reorganization of systems related to nociception - enhanced nociceptive input, reduced descending inhibition, and/or descending facilitation - or whether the key systems are extra-nociceptive, and the pain is generated and maintained in the brain itself. The present study offers findings that are broadly consistent with both hypotheses. Of course, it’s difficult to say much more, because the relationship of these systems with either evoked pain experience or chronic, ongoing back pain intensity was not studied here - and the resolution of the regions that are the units of analysis in current ‘modularity’ analyses is too coarse to say for sure what the relationship is between regional activity and either nociceptive or descending modulatory systems. Nonetheless, this paper is an important step forward.

 Other future challenges remain as well, of course, though this does not take away from the value of the present work. 

1. If we’re going to be able to count on measures as biomarkers for pain-linked systems, they’re going to have to be more accurate. They’ll also have to be linked to pain experience or other aspects of (dys)function, as well, and their specificity to pain (or other processes) will have to be assessed.
2. We need advances in how to deal with correlated variables in a multivariate predictive analysis framework. This is noted as a limitation here and some attempts were made to address selective confounds. But socioeconomic status, social standing, early-life adversity, body-mass/overweight, use of medicines and other drugs, sleep, and other variables are correlated with chronic pain incidence. If we think of these as confounds and try to look at the unique effects of back pain above and beyond all its various correlates and risk factors, we will never get anywhere. Mano et al. show that brain activity was predictive of chronic pain status, but not depression status (depressed vs. control). Other tests might be productively performed, including: Are the predicted chronic pain scores (e.g., distance from the SVM hyperplane) correlated with gender, depression, head movement, or other variables? And are they correlated with any of these more strongly than they correlate with chronic pain status? Mano et al. do report the [presumptive point-biserial] correlation of the pain classifier output with depression scores (r = 0.22), but do not compare this value with the correlation of the pain classifier output with pain. 
3. In the future, it will also be important for the field to develop strategies to effectively control for or minimize the influence of nuisance variables (here, gender and head movement displacement) in the course of SVM (or other) model training, identifying patterns optimized or constrained to be unrelated to them.  

Overall, this is a very nice effort, and it moves the needle in terms of advancing research on chronic pain.

I have read this submission. I believe that I have an appropriate level of expertise to confirm that it is of an acceptable scientific standard.

# Peer review: Referee response for version 1

**DOI:** 10.21956/wellcomeopenres.15303.r31759

**Published:** 2018-03-14

**Authors:** Apkar Vania Apkarian

**Affiliations:** 1 Departments of Surgery and Anesthesia, Northwestern University, Chicago, IL, USA

This is an elegant study, where the authors use 3 different resting state fMRI data in chronic back pain patients and controls, to test for brain network disruptions, and use such metrics to classify subjects using machine learning algorithms. 

 Using network properties and support vector learning algorithm, they demonstrate that patients and controls can be robustly differentiated from each other. The authors use two alternative classifications, sex and depression, as internal controls to test for outcome specificity. While the methods fails to differentiate sex, it was successful on depression. Here, I have some reservations. The depression classification was based on BDI score with a value = 3 used to define presence or absence of depression. It was not clear to me where this cut-off comes from. Certainly in the clinical literature this cut-off is meaningless, as it is too low. I wonder if this cut-off was increased to some value above 10, what the results would look like. In the same vein, it would be nice to see the actual correlation between pain and BDI, both in patients and healthy controls. Perhaps it would be more judicious, to do the classification after correcting one scale with the other. Otherwise, the results are always a mixture of both. See for example the study by Davis *et al*.(2016) ^1^. The same issue also applies to the classification using deep learning algorithm.

 The authors next test hub disruption using multiple network topographic measures. Interestingly, the one based on degree count, fails for the Japan data, although this measure was shown to be robust in our own hands, across humans and rodents, and for multiple chronic pain conditions. The observation is important from the viewpoint of across lab replicability of such measure, and the authors should be congratulated for taking such a step, especially in the field of pain research, where not enough is being done along replication. I want to note that they have already uploaded all of their fRMI data into OpenPain, and we are already using their data in a different research direction.

 A general point that may be important to address regards the specificity of identified networks both for classification and for modularity analyses. The authors emphasize somatosensory nodes as being the most important in the current analysis. In our earlier hub disruption study, we showed that removing the most significant nodes had no effect on hub disruption, and that we could randomly remove 99% of links and still see the same magnitude of disruption. It would be nice if the authors of the current study test the same idea. For example, in the SVM analysis, does classification fail when you remove the top links? Similarly, in the modularity analysis. Such an analysis has the potential of importantly changing the discussion viewpoint.

 Overall, although I raise points that can further expand on understanding reported results, this is a well done study with important conclusions, using state of the methods.

I have read this submission. I believe that I have an appropriate level of expertise to confirm that it is of an acceptable scientific standard.

## References

[1] DavisDAGhantousMEFarmerMABariaATApkarianAV: Identifying brain nociceptive information transmission in patients with chronic somatic pain. *Pain Rep*.2016;1(4) : 10.1097/PR9.0000000000000575 e575 10.1097/PR9.0000000000000575 28503674PMC5424698
